# Recent insights into the role of Akt in CD4 T-cell activation and differentiation: alternative splicing and beyond

**DOI:** 10.1097/IN9.0000000000000015

**Published:** 2023-01-23

**Authors:** Tristan L. A. White, Ye Jin, Matthew J. Gable, Penelope A. Morel

**Affiliations:** 1 Department of Immunology, University of Pittsburgh, PA, USA

**Keywords:** T helper cell, Akt, differentiation, alternative splicing, signaling

## Abstract

The activation and differentiation of CD4^+^ T cells is a complex process that is controlled by many factors. A critical component of the signaling pathway triggered following T-cell receptor (TCR) engagement is the serine threonine kinase Akt. Akt is involved in the control of many cellular processes including proliferation, metabolism, and differentiation of specific T_H_-cell subsets. Recent work has shown that, depending on the nature or strength of the TCR activation, Akt may activate different sets of substrates which then lead to differential cellular outcomes. Akt plays an important role in controlling the strength of the TCR signal and several recent studies have identified novel mechanisms including control of the expression of negative regulators of TCR signaling, and the influence on regulatory T cells (Treg) and T_H_17 differentiation. Many of these functions are mediated via control of the FoxO family of transcription factors, that play an important role in metabolism and Th cell differentiation. A theme that is emerging is that Akt does not function in the same way in all T-cell types. We highlight differences between CD4 and CD8 T cells as well as between Treg, T_H_17, and T_FH_ cells. While Akt activity has been implicated in the control of alternative splicing in tumor cells, recent studies are emerging that indicate that similar functions may exist in CD4 T cells. In this mini review, we highlight some of the recent advances in these areas of Akt function that demonstrate the varied role that Akt plays in the function of CD4 T cells.

## 1. Introduction

Naive CD4^+^ T cells are induced to differentiate into specific effector T helper (T_H_) cells following recognition of their cognate antigen presented by antigen presenting cells (APCs), such as dendritic cells (DCs). These T_H_-cell subsets include T_H_1, T_H_2, T_H_17, T_H_9, T follicular helper (T_FH_) and regulatory (Treg) cells, and are characterized by the expression of specific master transcription factors (TFs) and the secretion of unique sets of cytokines ^[1]^. Many factors play important roles in determining which TH-cell subset differentiates in response to the first antigen encounter ^[2,3]^, including the strength of the T-cell receptor (TCR) signal, cytokines produced by DCs and other APCs, as well as costimulatory molecules expressed by the APC.

TCR engagement leads to the activation of many signaling pathways downstream of the TCR. One of the most studied is the PI3K/Akt/mTOR pathway ^[4]^, which has a major influence of T_H_ cell differentiation ^[5–7]^. Upon activation of T cells multiple signaling cascades lead to the recruitment of PI3K to the membrane ^[4]^. The conversion of PIP_2_ to PIP_3_ by PI3K results in Akt re-localization to the membrane via its PH domain. Full activation of Akt requires phosphorylation on threonine 308 (T308) by phosphoinositide-dependent protein kinase 1 (PDK1) ^[8]^ and on serine 473 (S473) by mechanistic target of rapamycin complex 2 (mTORC2) ^[9]^. The precise mechanism by which Akt is fully activated in T cells is not fully clarified. Earlier studies demonstrated the importance of the PH domain of PDK1 in full activation of Akt ^[10]^ through its ability to bind PIP_3_ leading to T308 phosphorylation. A more recent study showed that TCR signal strength influences the relative abundance of PIP_2_ and PIP_3_^[11]^. High TCR signal strength was required to generate high levels of PIP_3_, which led to the activation of both PDK1 and mTORC2, and dual Akt phosphorylation, whereas low TCR signal strength favored the production of PIP_2_, and this was shown to influence T_H_ cell differentiation toward Treg ^[11]^. Akt, a serine/threonine kinase, influences many cellular processes via the phosphorylation of downstream substrates including metabolism, TF localization, and alternative splicing (AS).

Early studies, including our own, showed that full activation of Akt, as evidenced by phosphorylation at both T308 and S473 sites, inhibited the differentiation of Treg cells ^[12–14]^. Furthermore, we demonstrated that activation of T cells with low TCR signal strength leads to the partial phosphorylation of Akt, at T308, resulting in changes in the substrates that are phosphorylated by Akt ^[15]^. The precise mechanisms by which Th cell differentiation is influenced by the activation states of Akt are not fully understood. In this mini review, we explore the recent literature that sheds light on these potential mechanisms.

## 2. Early TCR Signaling Influences Akt Activity

Negative regulators play important roles in TCR signaling by controlling Akt activity and function. Our studies focused on the role of the lipid phosphatase PTEN ^[16]^, but recent studies have identified additional negative regulators of Akt activation and function ^[17–19]^. A recent study ^[19]^ implicated Akt in the control of Cbl-b protein expression, a E3 ubiquitin ligase known to negatively regulate TCR signaling. It was reported that haploinsufficiency of *Cbl-b* resulted in higher susceptibility to autoimmunity in a murine model. The stability of the Cbl-b protein was shown to be positively regulated via two phosphorylation sites by GSK3, which, in turn, is inactivated by Akt activity. Further, it was shown that constitutive Akt activation led to a loss of tolerance associated with a loss of Cbl-b expression ^[19]^.

Protein tyrosine phosphatase SHP-1-deficient CD4^+^ and CD8^+^ T cells were shown to resist Treg-mediated suppression in a T cell-intrinsic manner ^[18]^. The data also suggested that such resistance to Treg suppression was mediated by the enhanced phosphorylation of Akt on T308 in SHP-1 null T cells ^[18]^. Another group studied the impact of protein phosphatase 6 (Pp6) on Tregs by utilizing *Pp6*^*fl/fl*^x*FoxP3*^*cre*^ mice ^[17]^. They reported that conditional deletion of *Pp6* in Tregs exacerbated experimental colitis and experimental autoimmune encephalomyelitis (EAE) due to reduced *FoxP3* expression and impaired immunosuppressive function of Tregs. Their results suggested that diminished FoxP3 expression in Tregs was mediated by increased CpG methylation of the *FoxP3* locus and enhanced Akt phosphorylation on both T308 and S473 ^[17]^. In contrast, a recent study in human Th cell differentiation demonstrated the role of PD-1 ligation in the conversion of effector T cells into induced Tregs through attenuated Akt/mTORC1/S6 signaling ^[20]^. These studies indicate that Akt activity negatively correlates with the induction and/or function of Tregs at the level of early TCR signaling.

## 3. Akt and T-cell expansion and differentiation

Recent studies reported the involvement of Akt activity in T-cell expansion. These studies showed that CD8^+^ T cells lacking interferon regulatory factor 4 (IRF4) had reduced homeostatic proliferation, that was associated with increased PTEN expression. This led to a reduction in Akt signaling and activity, and demonstrated an important role for IRF4 in controlling PTEN expression ^[21]^. In contrast, long noncoding RNA *Morrbid*, induced following activation of LCMV-specific CD8^+^ T cells in vivo, was shown to negatively regulate the Akt/mTOR pathway leading to a reduction in CD8^+^ T-cell expansion and effector function ^[22]^.

Akt activity has been shown to affect T-cell differentiation. A comprehensive study of how PI3K p110δ controls CD8^+^ T cell fate, demonstrated that PI3K p110δ controlled many important functions of naïve and effector CD8^+^ T cells, some of which were also dependent on Akt activation ^[23]^. In particular, the production of multiple PI3K p110δ-dependent TCR-induced cytokines and chemokines required Akt activity ^[23]^. Interestingly, studies of human T_H_17 differentiation showed that CD28 co-stimulation inhibited T_H_17 differentiation ^[24]^. This was associated with increased Akt activation and the addition of Akt inhibitors in the presence of CD28 co-stimulation enhanced T_H_17 differentiation. Thus, an intermediate level of Akt activity was most effective for IL-17 production in the presence of CD28 co-stimulation, suggesting that the tuning of Akt activation is of importance in modulating CD4^+^ T-cell differentiation programs ^[24]^. A more recent study showed that CD28 stimulation induced CTLA-4 expression on human T_H_17 cells, but not T_H_1 cells, in an Akt-dependent manner, as demonstrated by the use of Akt inhibitors ^[25]^. Furthermore, overexpression of FoxO1 or FoxO3 repressed CTLA-4 expression in T_H_17 but not T_H_1 cells, demonstrating the connection between Akt activity and CTLA-4 expression through FoxO1 and/or FoxO3 ^[25]^.

## 4. Akt and Metabolism in Activated T Cells

Following activation, T cells undergo rapid changes in metabolism and this may vary depending on the T_H_ cell subset ^[26]^. Recent studies reported that, under low TCR conditions, Akt activation resulted in the phosphorylation of citrate synthase, thereby inactivating it. This resulted to increased nuclear acetyl-CoA levels, which led to increased histone acetylation at the *FoxP3* promoter and thus the induction of *FoxP3* transcription ^[27]^. The early glycolytic response in human CD4^+^ T cells following stimulation is dependent on Akt activity. However, the Akt pathway is dispensable for the induction of glycolysis in murine CD8^+^ T cells, indicating the different requirement for Akt activity in rapid activation-induced glycolysis between CD4^+^ and CD8^+^ T cells ^[28,29]^.

## 5. Akt and the regulation of FoxO1/FoxO3 activity

In quiescent T cells FoxO1 and FoxO3 are in the nucleus and control the expression of multiple genes important for T-cell metabolism and function ^[30,31]^. Upon T-cell activation and the phosphorylation of Akt at both T308 and S473 sites, Akt directly phosphorylates FoxO1 at Thr24 ^[32]^, leading to its relocation to the cytosol ^[31]^ where it can no longer act as a TF ^[16,33]^. Several groups observed that after pFoxO1 leaves the nucleus, it binds 14-3-3 and becomes ubiquitinated prior to proteasomal degradation ^[34–36]^. The degradation and elimination of FoxO1 is transient, and after 24 hours of in vitro stimulation, FoxO1 is highly expressed and again located in the nucleus ^[37]^. It has also been shown that FoxO1, along with other factors such as EOMES and Tcf-1 are necessary to support long-lived memory T cells ^[38,39]^.

FoxO1 is important for PI3K/Akt-dependent T_FH_ differentiation. These studies showed that signaling via the costimulatory molecule ICOS led to FoxO1 inactivation by the Akt-dependent mechanism described above ^[37,40,41]^. Furthermore, it was shown that the E3-ubiquitin ligase Itch was necessary for the degradation of FoxO1 during T_FH_ differentiation ^[41]^. In fact, T cells with a specific deletion of FoxO1 stimulated for 4 days led to the entire population expressing CXCR5^int^, a characteristic marker for T_FH_ cells ^[37]^.

In scenarios of low-dose stimulation in which Akt is not dually phosphorylated, FoxO1 is not phosphorylated and remains in the nucleus ^[16]^. T cells stimulated with low doses of antigen in vitro will develop a Treg phenotype because FoxO1 is necessary for Foxp3 transcription ^[30,42,43]^. In addition, inhibition of Akt in CD4 T cells stimulated with a high dose of antigen prevents FoxO1 phosphorylation and it is retained in the nucleus ^[16]^. Mice with a conditional knock out of FoxO1 in Treg demonstrated severe autoimmunity with a loss of Treg function ^[31]^, demonstrating the importance of FoxO1 in the induction and maintenance of Treg.

Recently, the Akt phosphorylation sites on FoxO1 were specifically mutated in T cells to Alanine, thereby preventing its inactivation following T-cell activation ^[44]^. These mice showed stunted growth, splenomegaly, and enlarged lymph nodes. There was a significant decrease in the total number of CD4 T cells. Constitutive FoxO1 activity led to drastic metabolic changes within the cell, such as decreased glucose consumption and lactate production, as well as mitochondrial deficiencies, which ultimately decreased cell proliferation and survival. These defects were shown to be caused by impaired Myc/IL2 signaling. CD25 levels were unchanged, but IL-2Rb (CD122) levels were decreased at baseline and became progressively lower after activation. Interestingly, these mice demonstrated increased Akt activity that was independent of PDK1 and mTORC2 and appeared to be mediated by a non-canonical pathway. These studies demonstrate the importance of the kinetic control of Akt and FoxO1 activation in the activation and proliferation of T cells ^[44]^.

## 6. Akt and the regulation of AS

AS is the complex process by which different messenger RNA species are produced following the deletion or retention of exons, leading to the translation of different protein isoforms ^[45,46]^. Sequencing of the human genome and transcriptome has revealed that over 90% of multi-exon genes are alternatively spliced in a tissue-specific manner ^[45,46]^. AS is regulated by RNA-binding protein (RBP) activity ^[47,48]^, many RBPs are known to control AS in immune cells ^[47,48]^, and there is increasing evidence that Akt controls the function of these proteins ^[15,49]^.

Akt activity has been shown to affect AS in various cancer models ^[50–52]^ through actions on caspase 9 ^[51,52]^ and RNA processing regulator IWS1 and FGFR-2 ^[50]^. Our own studies identified RNA processing factors, including hnRNP A1 and hnRNP L, as Akt targets during T_H_ cell differentiation ^[15]^. A recent study examined the role of AS and RNA processing in Treg induction and stability ^[49]^. This study showed that inhibition of PKCθ during in vitro Treg differentiation altered AS patterns by affecting the activity of the RNA processing factor hnRNP L and the methyltransferase PCMT1, leading to greater stability of Treg ^[49]^. While this study did not directly examine the role of Akt, the authors proposed that it could be playing a role in controlling PCMT1 function, and further studies are needed to confirm this speculation. A recent study demonstrated a role for the PI3K/Akt pathway in the IFN-γ-induced AS of PD-1 in human monocytes ^[53]^. The alternatively spliced PD-1 was shown to play a role in T-cell regulation and blockade of Akt, PI3K, and Jak moelcules inhibited the generation of this splice variant ^[53]^.

AS plays an important role in thymic T-cell development. The expression of Foxa1 and Foxa2 is important for the development of CD4 and CD8 T cells by regulating the expression of splicing factors and regulators ^[54]^. Loss of Foxa1 and Foxa2 during the double positive stage of T-cell development in the thymus leads to over 850 differentially used exons and a reduction of T cells in the periphery. This study showed that Foxa1 and Foxa2 regulated the expression of multiple splicing factors and regulators including hnRNP A1 ^[54]^. hnRNP A1 has been implicated in the splicing of Foxp3 during Treg development ^[55]^, and we have shown that hnRNP A1 knockdown inhibits Treg induction in vitro ^[15]^.

Another RBP important in T cell development is hnRNP L which has been implicated in the AS of CD45 and many other T cell signaling molecules ^[49,56]^. Mice in which hnRNP L is deleted early in T-cell development demonstrate a profound defect in T-cell development associated with changes in T-cell signaling and a migration defect from the thymus ^[57]^. This was associated with multiple changes in AS, including CD45 ^[57]^. A recent study examined the role of AS in T-cell activation and differentiation ^[58]^ by analyzing multiple RNA-seq datasets from naive CD4 T cells and T_H_ subsets. The study identified close to 1200 genes undergoing AS and analysis of the expression profiles of RBPs and their cognate binding sites flanking the discovered AS events revealed several RBPs associated with these events including hnRNP A1 and hnRNP L ^[58]^. Many of these RBPs were found to be T_H_ subset specific, for example, the conserved binding sites flanking AS events in T_H_17 cells were hnRNP A1/hnRNPA2B1.

The precise connection between Akt activity and how these splicing events occur through specific RBPs requires further work, but these studies suggest that progress is being made in this area.

## 7. Conclusions

The differentiation of naive CD4 T cells into specific T_H_ cell subsets is a highly complex process involving many important factors. This review focused on recent advances in our understanding of how the serine/threonine kinase Akt influences this process. Akt plays a central role in many cellular processes including T-cell activation, TF function and localization, metabolism, and AS. In general, full Akt activation is required for effector T_H_ cell differentiation, whereas partial Akt function favors Treg induction. Understanding the function and regulation of important factors such as Akt in T_H_ cell differentiation and function will open new potential therapeutic targets in such fields as cancer immunotherapy and autoimmunity.

## Conflicts of interest

The authors declare no conflicts of interest.

## Funding

This work was supported by NIH grants R01 AI125513 (PAM) and F31 AI152320 (TLAW).

## Acknowledgments

TLAW, YJ, MJG, and PAM wrote the article and provided editorial comments. PAM had primary responsibility for the final content. All authors have read and approved the article.
